# Applications of CRISPR/Cas System to Bacterial Metabolic Engineering

**DOI:** 10.3390/ijms19041089

**Published:** 2018-04-05

**Authors:** Suhyung Cho, Jongoh Shin, Byung-Kwan Cho

**Affiliations:** 1 Department of Biological Sciences and KI for the BioCentury, Korea Advanced Institute of Science and Technology, Daejeon 305-701, Korea; joshn@kaist.ac.kr; 2 Intelligent Synthetic Biology Center, Daejeon 305-701, Korea

**Keywords:** CRISPR/Cas, CRISPRi, CRISPRa, genome editing, gene regulation, metabolic engineering

## Abstract

The clustered regularly interspaced short palindromic repeats/CRISPR-associated (CRISPR/Cas) adaptive immune system has been extensively used for gene editing, including gene deletion, insertion, and replacement in bacterial and eukaryotic cells owing to its simple, rapid, and efficient activities in unprecedented resolution. Furthermore, the CRISPR interference (CRISPRi) system including deactivated Cas9 (dCas9) with inactivated endonuclease activity has been further investigated for regulation of the target gene transiently or constitutively, avoiding cell death by disruption of genome. This review discusses the applications of CRISPR/Cas for genome editing in various bacterial systems and their applications. In particular, CRISPR technology has been used for the production of metabolites of high industrial significance, including biochemical, biofuel, and pharmaceutical products/precursors in bacteria. Here, we focus on methods to increase the productivity and yield/titer scan by controlling metabolic flux through individual or combinatorial use of CRISPR/Cas and CRISPRi systems with introduction of synthetic pathway in industrially common bacteria including *Escherichia coli*. Further, we discuss additional useful applications of the CRISPR/Cas system, including its use in functional genomics.

## 1. Introduction

The clustered regularly interspaced short palindromic repeats (CRISPR)-associated (Cas) system, known as the prokaryotic adaptive immune system, is present in bacteria and archaea [1,2]. Owing to the mechanistic understanding of the response of the system to exogenous DNA, the CRISPR/Cas9 system has rapidly advanced in precise RNA-guided genome engineering in various cellular systems. Unlike previous genome engineering technologies, including zinc finger nucleases (ZFNs) and transcription activator-like effector nucleases (TALENs) [3,4,5], the CRISPR/Cas9 system allows for efficient modification of the desired genomic target by simply replacing 20-nt sequences of a chimeric single-guide RNA (sgRNA) complementary to the target sequence of interest. In addition, the nuclease-deactivated Cas9 (dCas9), which only has DNA-binding function guided by sgRNA, has demonstrated the potential to control regulatory functions in gene expression [6,7,8]. Along with its numerous therapeutic applications in eukaryotic systems, the CRISPR/Cas9 system has also been applied to engineer bacterial cells for industrial purposes. This review discusses the applications of the CRISPR/Cas9 system for metabolic engineering of bacterial cells to manipulate cellular functions such as increasing the yield and productivity of targeted value-added biochemicals. 

## 2. CRISPR/Cas9 System

**Figure 1:**
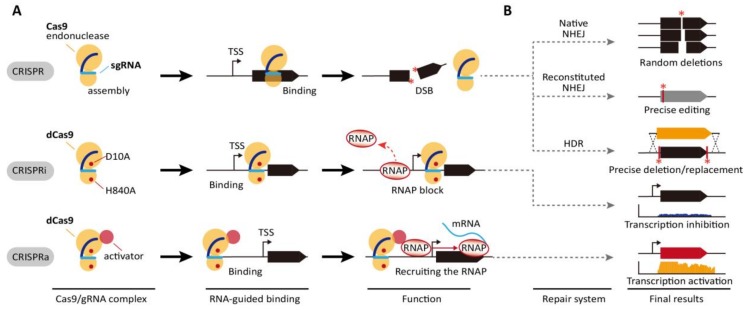
The CRISPR, CRISPRi, and CRISPRa systems. (**A**) CRISPR/Cas system consists of a Cas9 protein and a designed chimeric sgRNA complementary to the genomic target sequence (blue line). Upon the binding to the specific DNA sequence by Cas9-sgRNA complex, the DNA is cleaved by Cas9 with endonuclease activity. CRISPR interference (CRISPRi) is caused by a catalytically dead Cas9 (D10A and H840A mutations indicated by red dots), denoted as dCas9. The dCas9-sgRNA complex binds to the upstream region of the gene of interest, resulting that the process of RNA polymerase (RNAP) is inhibited and consequently transcription is blocked. CRISPR activation (CRISPRa) is applied for gene activation by the fusion of dCas9 and transcription activators such as RNAP ω subunit in *E. coli*. (**B**) Double strand break formed by CRISPR/Cas system can be repaired by the error prone non-homologous end joining (native NHEJ) pathway, resulting in the formation of random sized deletions around the targeted DNA sequence. With expression of a ligase such as LigD, NHEJ pathway creates the precise editing (reconstituted NHEJ). With the presence of homologous template, the gene deletion or replacement by homology-directed repair (HDR) is generated with high efficiency and the precision with near 100% frequency [8,19]. The asterisk indicates DSB-dependent modified region.

In principle, pre-CRISPR RNAs (pre-crRNA) transcribed from clusters are first processed to a single spacer and repeated units. Processed crRNAs hybridize with protospacer target DNA along with Cas endonuclease, thereby producing a double-stranded break (DSB) in the target sequence (Figure 1A). CRISPR/Cas systems are classified as Class 1 and Class 2, which include a complex of multiple Cas proteins and a single large Cas protein, respectively, and further divides into various subtypes based on the their complexity and signature proteins [9]. The type II CRISPR/Cas9 and type V CRISPR/Cas12a (Cpf1) in the Class 2 have been extensively used for genome engineering across bacterial, plant, and eukaryotic cells [10,11,12,13]. The most widely used type II CRISPR/Cas9 system from *Streptococcus pyogenes* consists of two components for its function: Cas9 nuclease and the single-guide RNA (sgRNA) modified from the crRNA: trans-activating crRNA (tracrRNA) duplex [14]. Its simple and highly specific cleavage at the target sequence has yielded significant advancements in the development of versatile tools for editing and manipulating genomes in various organisms [15,16]. Cas9 induces site-specific DSBs through cleavage to the 20-bp sgRNA by the histidine-asparagine-histidine motif (HNH) nuclease domain and to the other DNA strand by the RuvC domain [17]. DSB induced by Cas9 then enables the modification of a target gene sequence through the non-homologous end joining (NHEJ) repair pathway or the homology-directed repair (HDR) system (Figure 1B) [18].

Furthermore, H840A and D10A substitutions in Cas9 inactivate the endonuclease function of the HNH and RuvC domains, respectively. The two mutations direct the formation of a functionally modified nuclease with deactivated Cas9 (dCas9), which can only bind target genomic loci directed by sgRNA [17]. This system has been intensively repurposed for genome engineering, metabolic engineering [20], epigenetic modification [21], functional genomics [11], genome imaging [22], and DNA/RNA chromatin immunoprecipitation [23]. In particular, dCas9 is a powerful tool for regulating transcription levels of a target gene. The binding of dCas9 directed by gRNA to the specific genomic locus can efficiently inhibit the progress of RNA polymerase (RNAP) to the downstream gene. Thus, this simple interference system known as CRISPRi enables the regulation of expression of target genes transiently or constitutively. In bacterial cells, CRISPRi is the more preferable genetic engineering tool for gene knock-down than RNAi (Figure 1A) [24,25]. 

In addition to simple transcriptional interference, dCas9 is often fused with various transcriptional repressors to maximize interference efficiency. dCas9 has been fused with the myc-associated factor X (MAX)-interacting proteins 1 (MXI1), a transcriptional repressor domain interacting with the histone deacetylase Sin3 homolog in yeast, the Krüppel-associated box (KRAB) domain of Kox1, a protein promoting heterochromatin formation, the chromo shadow (CS) domain of HP1α, a protein related in H3K9me3-dependent gene silencing, and the hairy-related basic helix-loop-helix repressor proteins (WRPW) domain of Hes1 [26,27,28,29,30]. These modified dCas9s demonstrated more efficient and robust gene inactivation than the simple interference system. In addition, the fusion of transcriptional activator to dCas9, called CRISPRa, induces transcription via recruitment of RNAP (Figure 1A). In eukaryotic cells, the herpes simplex VP16 activation domain and the nuclear factor κB (NF-κB) p65AD activation domain have been fused to dCas9 [28,31,32,33,34,35]. Transcriptional activation levels can be increased through tandem fusion of the domains with dCas9 [36,37,38,39]. In bacteria, dCas9 fused with the RNAP ω subunit was reported to activate the expression of a gene up to three folds [40]. 

## 3. Application of CRISPR/Cas9 Technology to Engineer Bacterial Genomes

The CRISPR/Cas9 system has been used in the various bacterial cells, through utilization of their genetic manipulation tools for Cas9 expression and gRNA, such as *Bacillus*, *Clostridium*, *Corynebacterium*, *Escherichia coli*, *Lactobacillus*, *Mycobacterium*, *Pseudomonas*, *Staphylococcus*, and *Streptomyces*. Here, we discuss the application of CRISPR/Cas9 or CRISPRi system in various bacterial cells and how the CRISPR/Cas system has been utilized for industrial purposes (Table 1).

### 3.1. Bacilli

*Bacilli* are industrially important for their ability to synthesize various proteins and biochemicals. In *B. subtilis*, the type II CRISPR/Cas9 system was applied for genome editing using a single plasmid system harboring pUC origin of replication and temperature-sensitive replication origin pE194^ts^ [43]. This system successfully yielded two large deletions from the *B. subtilis* chromosome: a 25.1-kb region containing *amyE* gene and a shorter 4.1-kb deletion containing the pulcherrimin biosynthesis genes and the repaired *trpC2* gene mutation. For efficient selection of genome-edited cells, the CRISPR/Cas system was expressed in the plasmid harboring a temperature-sensitive origin of replication, which is quite useful. Since homologous recombination in *B. smithii* occurs at 45–55 °C, counter-selection of the non-edited cells can be achieved through DSB formation at 37 °C and Cas9 inactivation at 42 °C. This system enables temperature-regulated gene deletion, gene knockout with insertion of premature stop codons, and gene insertion [41]. Further, the CRISPR/Cas9 system (ThermoCas9) controllable in a broader temperature range (20~70 °C) was developed in *Geobacillus thermodenitrificans* T12 and its protospacer adjacent motif (PAM) sequence preference was characterized in accordance with temperature variations [45]. In *B. licheniformis*, *yvmC* was deleted with 100% efficiency, using CRISPR/Cas9 nikcase [46]. This system showed that multiplexed gene deletion was achieved with an efficiency of 11.6% and large DNA fragment (42.7 kb) deletion with an efficiency of 79.0%. In the case of gene insertion, the efficiency was 76.5%. 

Despite advancements in the CRISPR/Cas9 system in bacterial cells, genome editing is not always successful for the bacterial genomes. As indicated previously, DSBs induced by CRISPR/Cas9 systems can be repaired via homologous recombination (HR) or NHEJ mechanisms (Figure 1B). Both HR and NHEJ are active for DSB repair pathways in eukaryotic cells. However, bacterial cells rely on HR and NHEJ has been limited for specific bacterial cells such as *Mycobacterium*, *Pseudomonas*, *Bacillus*, and *Agrobacterium*, which possess two core proteins, Ku and a ligase such as LigD [90,91]. Moreover, NHEJ repair in bacteria is often mutagenic, resulting in approximately 50% error rate typically because of a single nucleotide insertion [92]. Interestingly, the NHEJ repair system in *B. subtilis* does not co-occur with the type II-A CRISPR/Cas9 system [42]. This suggests that the activity of type II-A CRISPR/Cas9 was inhibited by the limited access of Ku by binding of Csn2 at the DNA ends. Instead, type II-C systems without Csn2 had no strong avoidance of co-occurrences with NHEJ [93]. 

### 3.2. Clostridia

*Clostridia* are anaerobic, Gram-positive, and spore-forming bacilli, which cause several significant pathogenic responses such as antibiotic-related diarrhea and produce multiple toxins such as Toxin A (TcdA) and Toxin B (TcdB) in *C. difficile* and Botulinum toxin in *C. botulinum* [94,95,96]. In particular, the toxin in *C. botulinum* is the most potent biological fetal poison which paralyzes muscles; however, this toxin is currently widely used for cosmetic surgery and numerous therapeutic purposes including the management of different muscle disorders and headache [97]. With such rapidly growing demands, the development of genome manipulation technology is also required for industrial application of *Clostridium* species. In *C. botulinum* strains, numerous CRISPR arrays were recently reported with a remarkable frequency in plasmids (80%) [48]. Those were type I or III CRISPR/Cas systems, not type II, and the spacers displayed homology with bacterial plasmids. The CRISPR/Cas9 system was first applied to solventogenic *clostridia*, such as *C. beijerinckii* and *C. acetobutylicum*, with industrial significance for the production of biofuels and biochemicals such as acetone, butanol, and ethanol (ABE). However, engineering of these bacteria is limited by the lack of genetic tools [98,99]. Despite a very low homologous recombination efficiency, CRISPR/Cas9-assisted homologous recombination enabled markerless gene deletion in *C. beijerinckii*, including the large gene fragment deletion (1.5 kb) from its chromosome, and integration and single nucleotide modification with high efficiency [55,100]. Furthermore, a CRISPRi system was successfully used to repress *spo0A* expression in *C. beijerinckii* [53]. In *C. acetobutylicum*, highly accurate genome modifications, nucleotide substitution, deletion, and integration of large genes up to 3.6 kb were achieved using a two-plasmid inducible CRISPR/Cas9 system [49]. As CRISPR/Cas9 system was also applied to a hyper-butanol-producing *C. saccharoperbutylacetonicum* N1-4, the highest butanol yield (19.0 g/L) from batch culture was obtained using the double deletion mutant of the *pta* and *buk*, which are essential genes for acetate and butyrate production [50].

### 3.3. Corynebacteria

**Figure 2:**
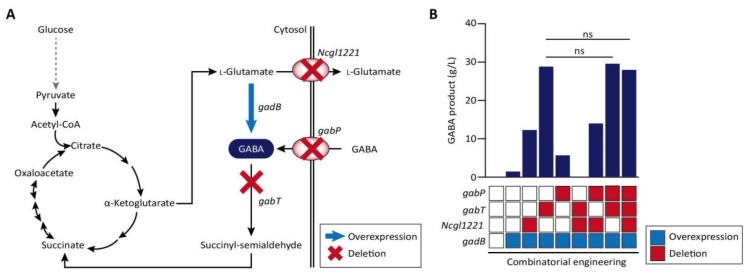
A metabolic engineering strategy for GABA production in wild-type *C. glutamicum*. (**A**) Metabolic pathway for GABA production in *C. glutamicum*, where genes *Ncgl*1221, *gabT* and *gabP* encoding *the* l-glutamate transporter, GABA transaminase and GABA permease, respectively, are targeted for deletion, using CRISPR/Cas, and *gadB* is overexpressed. Dotted arrow indicates simplified schematic of glycolysis pathway. The enzymatic reactions are represented by two-way or single black arrows, according to the reversibility of the reactions. (**B**) Through combinatorial gene regulation of four genes, the key gene for GABA production was screened [59].

*Corynebacteria* are Gram-positive bacteria with a high GC content and very useful for the production of biomaterials. In particular, *C. glutamicum* is the most well developed strain among *Corynebacteria* for the industrial production of amino acids and also has no endotoxins and has minimal protease activity and an efficient protein secretion system [58,101]. In several cases, type II CRISPR/Cas9-based genome manipulation was achieved for gene deletion, point mutation, and insertion in *C. glutamicum* genome with high efficiency (up to 100%). The disruption of the *porB*, *clpX*, and Ncgl0911 genes in *C. glutamicum* affected protein expression, and reporter green fluorescence protein (GFP) was more upregulated in the deletion mutant compared to the wild-type strain. Liu et al. also reported the performance of CRISPR/Cas9 system for gene deletion (up to 60.0%), insertion (62.5%), single-nucleotide editing (90%), and double-locus editing [57]. The CRISPR/Cas9-coupled homologous recombination system was further engineered with the RecT recombinase, which is used to incorporate synthetic single-stranded oligodeoxyribonucleotides into the genome for the production of γ-aminobutyric acid (GABA) [59,102]. Through combinatorial gene knockouts of *Ncgl*1221, *gabT*, and *gabP* genes coding l-glutamate exporter, GABA transaminase, and GABA permease, respectively (Figure 2A), the productivity of GABA was enhanced up to 28.7 g/L in all knock-out mutants (Figure 2B). Knock-down of *gabT*, which degrades GABA for recycling in the tricarboxylic acid (TCA) cycle, seems to block the conversion of GABA to succinyl-semialdehyde in the cytosol. CRISPRi-based gene regulation in *C. glutamicum* was also performed for amino acid production. The repression of *pgi* leads to NADPH overproduction through the pentose-phosphate pathway, increased l-lysine titers, and the repression of *pck* and *pyk* is known to increase l-glutamate production [103,104,105]. Multiplex knock-down of *pgi* and *pck* up to 98%, and of *pyk* up to 97% increases l-lysine and l-glutamate production comparable to levels achieved via gene deletion [60].

### 3.4. Streptomycetes

As the largest genus of Actinobacteria, *Streptomyces* strains are Gram-positive bacteria with a high GC content and abundant in soil and produce numerous clinically and industrially important secondary metabolites [106,107]. The representative secondary metabolites are antibiotics such as vancomycin from *S. griseus*, herbicides such as phosphinothricin from *S. hygroscopicus*, chemotherapeutics such as daunoarubicin from *S. peucetius*, and immunosuppressants such as rapamycin from *S. hygroscopicus* [19,108,109]. For the identification, characterization, engineering, and production of the secondary metabolites, efficient genetic manipulation tools are required; however, current genetic tools are still unsatisfactory with respect to their efficiency, which is a bottleneck for systematic metabolic engineering of *Streptomyces* strains [19,69]. 

**Figure 3:**
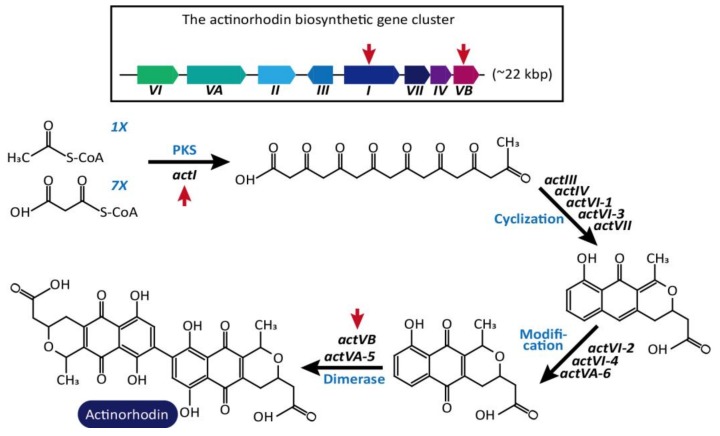
Regulation of actinorhodin biosynthetic pathway in *S. coelicolor* A3. The organization of the actinorhodin biosynthetic gene cluster (22 kbp) is shown. 1× Acetyl-CoA and 7× malonyl-CoA creates the long carbon skeleton by *actI*. Then, the carbon backbone is cyclized to form a (*S*)-DNPA by *actIII*, *actIV*, *actVI-1*, *actVI-3*, and *actVII*, and modified to DHK by *actVI-2*, *actVI-4*, and *actVA-6*. Two DHK molecules finally produce actinorhodin through dimerization by *actVB* and *actVA-5*. The biosynthesis of blue-pigmented polyketide antibiotic actinorhodin was inactivated by targeting *actI* and *actVB* by CRISPR/Cas9 [19] Red arrows indicate sgRNA targets.

Cobb et al. reported the use of the CRISPR/Cas9-based multiplex genome editing method for several Streptomyces strains, including *S. lividans*, *S. viridochromogenes*, and *S. albus* [69]. Using codon-optimized Cas9 from *Streptococcus pyogenes*, they constructed a pCRISPomyces plasmid harboring two sgRNAs and two editing templates for homologous recombination. This system demonstrated that targeted genomic regions of various sizes (ranging from 20-bp to 30-kb) could be deleted with up to 100% efficiency. In addition, Tong et al. developed a highly efficient CRISPR/Cas9 system for gene deletion, gene replacements, and reversible control of gene expression. As a model system, they used CRISPR/Cas9-mediated DSB to inactivate two genes, *actIORF1* (SCO5087) and *actVB* (SCO5092), in the biosynthetic pathway of the blue-pigmented polyketide antibiotic actinorhodin in *S. coelicolor* A3(2) and consequently, no blue pigment was observed (Figure 3) [19]. With the gene deletions, the insertion of fragments of random size, from a 1-bp up to over than 30,000-bp, was also examined around the DSB position by incomplete NHEJ pathway. Furthermore, the co-expression of the LigD ligase, a core component of NHEJ pathway from *Mycobacterium tuberculosis*, resulted in increased inactivation efficiency from 45 to 77% for ActIorff1-6 T and from 37 to 69% for Actvb-2NT. Further, the application of CRISPR/Cas9 with a homologous recombination template of ~2.2-kb yielded precise and high gene deletion and insertion efficiency of 90–100% without any harmful effect on cell growth. For regulation of the gene expression, the CRISPRi system targeting non-template strand of actinorhodin gene was also analyzed in *S. coelicolor*; actinorhodin biosynthesis was controlled by dCas9 expression levels.

**Figure 4:**
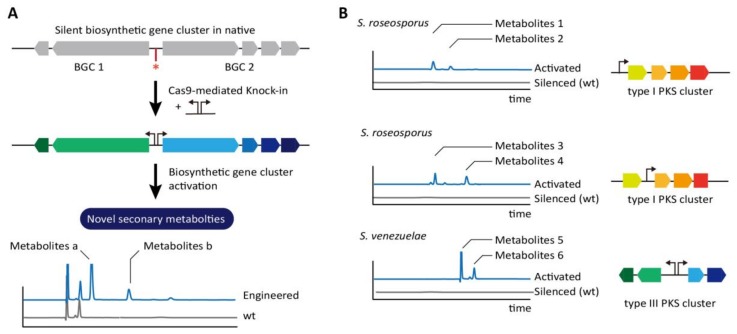
Identification of novel metabolites through insertion of synthetic promoter in the upstream of silent biosynthetic gene cluster (BGC) in native (**A**) Using CRISPR-Cas9, efficient and precise introduction of promoter cassettes (red-shaded arrows) induces the expression of biosynthetic genes and triggers the production of unique metabolites that are not detected in the wild-type strain. The asterisk indicates DSB-dependent modified region. (**B**) The metabolites of different type are shown according to the promoter insertion location in type I PKS cluster in *S*. *roseosporus*. In *S*. *venezuelae*, the insertion of a bidirectional promoter cassette between a type III PKS gene encoding an RppA synthase and a cytochrome P450 gene resulted in production of novel pigmented products [71].

To identify pharmaceutically valuable natural products from *Streptomyces* strains, the unknown biosynthetic gene clusters (BGCs) have been investigated. However, the majority of the secondary metabolites produced from BGCs are inadequate to be detected using current methods because of low expression levels of genes in BGCs and expressed only upon the specific environmental stresses [71]. A model system to trigger the production of unique metabolites by activation of those silent BGCs was designed on the basis of promoter replacement (Figure 4A). Strong and constitutive promoters were inserted using CRISPR/Cas9 with HDR upstream of the indigoidine cluster of *S. albus* and upstream of actinorhodin (ACT) and undecylprodigiosin (RED) clusters of *S. lividans*, which resulted in higher production of the pigments compared to the wild-type strain. Interestingly, the CRISPR/Cas9 system was also examined to activate a silent BGC from *S. roseosporus*, which shows relatively high homology to the known BGC of *S. venezuelae*. Promoter insertion upstream of the first open reading frame (ORF) in the cluster showing >90% sequence identity to the polycyclic tetramate macrolactam (PTM) in *S. lividans*, produced alteramide A, which is the original metabolite produced by *S. roseosporus*. Unexpectedly, an unknown second PTM having a simple planar structure similar to dihydromaltophilin were also detected, indicating the presence of host-dependent factors. Further, this activation strategy was applied to uncharacterized BGCs in *S. roseosporus* and *S. venezuelae* (Figure 4B). Activation of type II polyketide synthase (PKS) BGC in *S. viridochromogenes* yielded a novel brown pigment in liquid and solid media before sporulation. Thus, various applications of *Streptomyces* genome engineering based on the CRISPR/Cas9 can significantly enhance the mining of industrially and pharmaceutically important chemicals. 

### 3.5. Escherichia coli

Currently, *E. coli* is one of the most industrially significant platform strains for the production of various biochemicals and biofuels, including fatty acids, amino acids, polyketides, 1,4-butanediol, polyesters, and flavanones [110,111,112,113,114,115]. Advancements in metabolic engineering, systems biology, and synthetic biology have contributed to improve the productivity and yield of target products from *E. coli* [116,117,118]. In addition, *E. coli* is the most representative model system for developing new CRISPR/Cas tools with the highest efficiency of near 100% for precise genome modification including gene deletion and insertion [10,77]. In particular, CRISPRi is a useful tool to knock-down gene expression transiently or constitutively without disruption of the target gene. Upon consideration of metabolic pathways, CRISPRi can block the metabolic fluxes to by-products and increase the flux towards the synthesis of target products or to determine the direction for transport of target products by inhibiting transporter activity. Thus, the CRISPRi system can easily regulate gene expression levels by directing sgRNA to the region of non-template DNA of target genes [8]. For example, CRISPRi was applied to enhance flavonoid production by fine-tuning central metabolic pathways such as glycolysis, the TCA cycle, or fatty acid synthesis [76]. Malonyl-coenzyme A (malonyl-CoA), an important precursor metabolite for biosynthesis of flavanones [115], polyketides [114], and fatty acids, is the initial substrate for the synthesis of flavonoids [113,119]. Silencing of candidate genes increased intracellular malonyl-CoA levels by over 223%. Moreover, the titer of (2S)-Naringenin dependent on the amount of malonyl-CoA was increased to 421.6 mg/L, which was 7.4 folds higher than the control strain. Thus, CRISPRi system is a highly valuable tool for fine-tuning of metabolic pathways while minimizing the effects on cell growth [76]. 

β-carotene is a red-orange pigment belonging to the isoprenoid family and is used in pharmaceutical and nutraceutical industries. To enhance β-carotene production derived from isopentenyl pyrophosphate (IPP) and dimethylallyl pyrophosphate (DMAPP) into the methylerythritol phosphate (MEP) pathway across central metabolic pathways, the related metabolic fluxes were fine-tuned through a combination of gene deletions based on a CRISPR/Cas9 editing tool and gene overexpression [77,120]. Consequently, β-carotene (2.0 g/L) was produced from glucose as the sole carbon source. As another example, isoprenoids are naturally produced organic chemicals and classified in accordance with the number of 5-carbon (C5) isoprene units, which have been used commercially in fragrances, pharmaceuticals, insecticides, drugs, and biofuel [121,122,123,124]. Using the engineered *E. coli* harboring biosyntheticmevalonate (MVA) pathway and plant-derived terpenoid synthases, the CRISPRi system suppressed the expression of acetoacetyl-CoA thiolase enzyme catalyzing the first step in the MVA pathway [79]. This CRISPRi-guided balancing of expression of MVA pathway genes led to enhanced production of (−)-α-bisabolol (C15) and lycopene (C40) and alleviation of cell growth inhibition that may be caused by expression of multiple enzymes or production of toxic intermediate metabolites in the MVA pathway. In more cases, *E. coli* strains were further engineered to improve the productivity through redirection of metabolic flux, using the CRISPR or CRISPRi system. Mevalonate was increased by 41% by targeting *dnaA* and *oriC*, or by blocking nucleotide synthesis genes, *pyfF* or *thyA* [78].

For biofuel production, a controllable CRISPRi system was introduced with an array of sgRNAs to knock-down four endogenous genes (*pta*, *frdA*, *ldhA*, and *adhE*) for n-butanol production, involved in the formation of acetate, succinate, lactate, and ethanol, respectively, with a single, double, triple, and quadruple combinatorial targeting [85]. When heterologous gene clusters related with n-butanol biosynthetic pathways were introduced into *E. coli*, the yield and productivity were increased up to 5.4- and 3.2-fold, respectively. Furthermore, the biosynthesis of 1,4-butanediol (1,4-BDO) was optimized in *E. coli*, using a combination of CRISPR/Cas9 and CRISPRi systems [83]. Using the CRISPR/Cas system, the point mutation of *gltA*, replacement of native *lpdA* to heterologous *lpdA*, knock-out of *sad*, and insertion of large gene cassettes of 6.0 and 6.3-kb in length in the 1,4-BDO biosynthesis pathway, resulting in a titer of 0.9/L for 2 d. Further, the expression of *gabD*, *ybgC*, and *tesB* genes was suppressed by the CRISPRi system to avoid the process to succinate and gamma butyrolactone, resulting in 1.8 g/L titer. 

**Figure 5:**
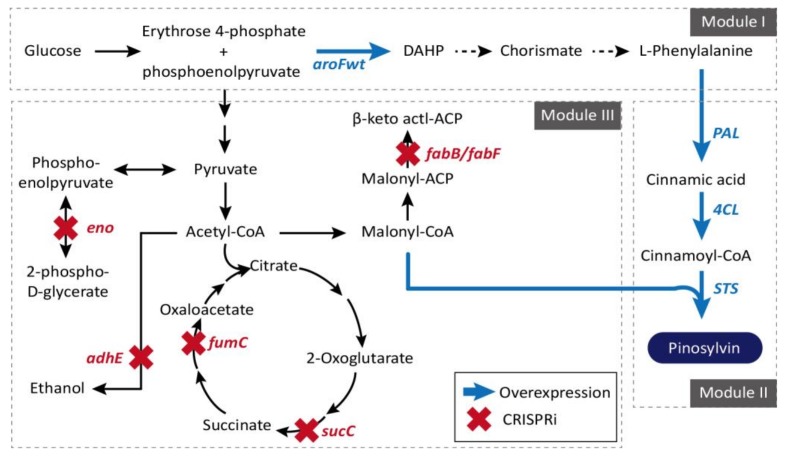
For high-yield pinosylvin synthesis, three different modules are selected and redirected. Genes selected for efficient channeling of the carbon flux toward malonyl-CoA, shown in red, are inhibited through CRISPRi. The metabolic pathway that performs heterologous biosynthesis of (2S)-naringenin from L-tyrosine in *E. coli* shown in blue was overexpressed. CHI: chalcone isomerase; CHS: chalcone synthase; 4CL: 4-coumarate:CoA ligase; E4P: erythrose-4-phosphate; PEP: phosphoenolpyruvate; TAL: tyrosine ammonia lyase [76].

Pinosylvin (*trans*-3,5-dihydroxystilbene) is a plant secondary metabolite activated by microbes or insects that is biosynthesized through a general phenylalanine metabolic pathway in genus *Pinus* [125,126]. Although a pharmaceutically promising anti-antioxidant, anti-cancer, cardioprotective, anti-inflammatory, and chemopreventive substance, its original productivity from plants is very low [81,127,128,129]. An efficient *E. coli* strain for pinosylvin production was developed through a rational modular design approaches [87]. Overexpression of the genes involved in module I (*aroF*^wt^ and *pheA*^fbr^, and TcPAL) and module II (4-coumarate:coenzyme A ligase (4CL) and stilbene synthase (STS)), and repression of module III genes (*fabB*/*fabF*, *adhE*, *eno*, *fumC*, and *sucC*) consisting of central metabolism were achieved using the CRISPRi system, resulted in final yield of 281 mg/L, which is the highest pinosylvin titer from d-glucose without any additional precursor supplements (Figure 5). More examples for the application of CRISPR/Cas9 and CRISPRi systems can be found from the efficient formation of poly(3-hydroxybutyrate-*co*-4-hydroxybutyrate) (P(3HB-*co*-4HB)) [80] and anthocyanins as glycosylated flavonoid pigments that are of significance in the food industry [82].

## 4. Bioinformatics Tools for Guide RNA Design in CRISPR/Cas9 System

Most sgRNA prediction softwares have been developed for eukaryotic systems, including CCTop [130], CHOPCHOP [131], CRISPR Design [132], CRISPRscan [133], E-CRISP [134], WU-CRISPR [135], WGE CRISPR Finder [136], CRISPy CHO [137], and GuideScan [138]. For bacteria, few softwares were currently available, such as sgRNAcas9 [139], CRISPy [140], CRISPR-ERA [141] and sgRNA scorer2.0 (https://CRISPR.med.harvard.edu/sgRNAScorerV2/) [142]. sgRNAcas9 software package supports a search for CRISPR target sites (protospacers) with user-defined parameters and predicts potential off-target cleavage sites (www.biootools.com). CRISPy-web is online resource to design sgRNAs for non-model organisms (http://crispy.secondarymetabolites.org/). This software enables to scan possible sgRNAs from the targeted genome of interest or partial gene excluding potential off-target matches. In addition, CRISPR-ERA is a sgRNA design tool for various organisms and this program generates sgRNA sequences for gene activation or repression by CRISPRi (http://CRISPR-era.stanford.edu). sgRNA scorer 2.0 enables to design sgRNAs for different Cas9 proteins from *S. pyogenes*, *S. aureus*, *N. meningitides*, *S. thermophiles* 1, and *S. thermophiles* 3 as well as the newly characterized CRISPR system Cpf1.

## 5. Concluding Remarks and Future Perspectives

In this review, we introduced the applications of CRISPR/Cas9 and CRISPRi systems in various bacterial cells for gene deletion, insertion, replacement, and regulation of gene expression. Although fine-tuning of metabolic flux is possible through rational design upon consideration of the known or predictable metabolic pathway, the results are not always successful to obtain the highest productivity or yield of target products, owing to the highly interconnected and complex metabolic network between core and branched metabolic pathways. Moreover, a rational design can control only the genes of known pathways by analyzing them individually, iterative deletion, combinatorial modification, or repression in several genes. In addition to the rational design, however, the engineered targets can be obtained through screening of the synthetic sgRNA library, which represents the whole set of bacterial genes. This efficiency of this approach can be accelerated by advancements in oligo library synthesis technology and detection methods such as high-throughput sequencing, fluorescence-activated cell sorting (FACS), and high-throughput microscopy. Recently, the CRISPRi system with synthetic gRNA library has been used for functional genomics studies in eukaryotic system, resulting in understanding of the functional roles of genes in diverse cellular mechanisms [143,144,145,146,147]. Similar to these examples, functional genomics using CRISPRi system was achieved in *B. subtilis* [44]. A CRISPRi library for the 289 known or proposed essential genes was constructed and used to examine changes in cell growth through gene knock-down. In particular, individual cellular responses were investigated in 93 conditions including various chemical treatments and oxidative stress at different doses. It enabled the identification of specific genetic responses to the treated conditions and revealed intimate genetic interconnected pathways in cell growth and in morphological changes through high-throughput microscopy. 

The innovative genome editing technology based on the CRISPR/Cas systems illustrates numerous advantages including ease of handling, high efficiency, and specificity, and variation in its applications regardless of the living organisms from prokaryotes to eukaryotes. Further future directions such as synthetic library-based screening methods have infinite potential such as modulation of platform organism for industrial use based on functional genomics. Since the CRISPR gRNA library size can deduct a more precise and comprehensive conclusion being accorded in the purpose, the development of highly efficient transformation methods is required. Alternatively, the library can be designed for the genes in the selected metabolic pathways or for regulatory parts such as promoters, ribosomal binding sites, and terminators. CRISPR/Cas has been used through constitutive or transient expression system according to the user’s purpose. For the knock-out or knock-down of specific genes, the constitutive expression systems can be preferred. However, transient expression is often useful to regulate the CRISPR/Cas system at the targeted time point. Moreover, in some bacteria, the CRISPR/Cas9 system is toxic to the cell and sensitive by the intracellular amount of Cas9 [148]. Therefore, the expression level of Cas9 protein needs to be controlled using inducible or tunable promoters [149,150]. In future, various controllable expression systems should be developed with the advance of CRISPR/Cas systems for a greater variety of industrial applications.

## Figures and Tables

**Table 1:** Applications of CRISPR/Cas9 or CRISPRi systems to bacterial genome editing and silencing.

Strains	Applications	Year	Ref.
***Bacillus***			
*B. smithii*	Genome deletion (90%), knockout (100%), insertion (20%)	2017	[41]
*B. subtilis*	DSB, non-homologous end-joining (NHEJ) repair	2017	[42]
*B. subtilis*	Deletion, Point mutation	2016	[43]
*B. subtilis*	CRISPRi library, Chemical genomics	2016	[44]
*B. smithii*	Genome editing and silencing, with ThermoCas9 (active at 55 °C)	2017	[45]
*B. licheniformis*	Genome deletion (1 kb) (100%), Two gene deletion (11.6%), Large gene deletion BacABC (42.7 kb) (79.0%), Gene insertion (75.5%)	2018	[46]
***Clostridium***			
*C. difficile*	Genome editing	2017	[47]
*C. botulinum*	Genome editing	2017	[48]
*C. acetobutylicum*	Gene substitution, Deletion, Insertion up to 3.6 kb	2017	[49]
*C. saccharoper -butylacetonicum*	Gene deletion (*pta*), Butanol production	2017	[50]
*C. beijerinckii*	CRISPRi	2016	[51]
*C. autoethanogenum*	Genome deletion	2016	[52]
*C. acetobutylicum*	CRISPRi, Genome deletion (20 bp)	2016	[53]
*C. beijerinckii*	CRISPRi, Genome deletion (20–1149 bp)	2016	[53]
*C. pasteurianum*	Genome editing	2016	[54]
*C. beijerinckii*	Genome editing	2016	[55]
***Corynebacterium***			
*C. glutamicum*	CRISPRi, *pyc*, *gltA*, *idsA*, *glgC*, *idsA*-*glgC*	2018	[56]
*C. glutamicum*	Genome deletion (60.0%), Insertion (62.5%), Modification (80%)	2017	[57]
*C. glutamicum*	Deletion (*porB*, *mepA*, *clpX* and *NcgI*0911), Insertion	2017	[58]
*C. glutamicum*	Genome deletion (up to 100%), Gamma-aminobutyric acid (GABA) production	2017	[59]
*C. glutamicum*	Genome editing (86–100%)	2017	[12]
*C. glutamicum*	CRISPRi (98%)	2016	[60]
***Lactobacillus***			
*Lactobacillus casei.*	Genome editing	2017	[61]
*Lactobacillus gassen*	17 strains, Optimization for CRISPR/Cas activity	2015	[62]
*Lactobacillus reuteri*	Site-directed mutagenesis (efficiency 90~100%)	2014	[63]
***Mycobacterium***			
*M. tuberculosis*	CRISPRi	2017	[64]
*M. tuberculosis*	CRISPRi	2016	[65]
***Psedomonas***			
*P. putida*	Genome editing, CRISPRi	2017	[45]
*P. aeruginosa*	CRISPRi	2018	[66]
*P. putida*	CRISPRi	2018	[66]
***P. fluorescens***	CRISPRi	2018	[66]
*Staphylococcus*			
*S. aureus*	Type III-A CRISPR/Cas system	2017	[67]
*S. aureus*	Genome deletion, Insertion, Single mutation	2017	[68]
***Streptomyces***			
*S. lividans*	Genome deletion (20 bp, 34 bp, 20–34 bp, 31,415 bp) (70 to 100%)	2015	[69]
*S. viridochromogenes*	Genome deletion (20 bp) (100%), 23 bp (67%)	2015	[69]
*S. albus*	Genome deletion (67 bp (100%) and 13214 bp (67%))	2015	[69]
*S. coelicolor A3*	Deletion (100%), CRISPRi	2015	[19]
*S. coelicolor A3*	NHEJ with LigD ligase coexpression	2015	[19]
*S. coelicolor A3*	HDR, Gene deletion	2015	[19]
*S. avermitilis*	Type I-E system	2016	[70]
*S. albus*	Knock in	2017	[71]
*S. lividans*	Knock in	2017	[71]
*S. roseosporus*	Knock in	2017	[71]
*S. venezuelae*	Knock in	2017	[71]
*S. viridochromogenes*	Knock in	2017	[71]
*S. rimosus*	Antibiotics (Oxytetracycline) production	2017	[72]
***Escherichia***			
*E. coli*	Programmable DNA looping (15%, 4.7 kb loop)	2017	[73]
*E. coli*	Point mutations, Deletions, Insertions, Gene replacements	2017	[74]
*E. coli*	CRISPR/Cas-assisted MAGE	2017	[75]
*E. coli*	CRISPRi, (2S)-naringenin, Carbon flux toward malonyl-CoA, (7.4 fold, 421.6 mg/mL)	2015	[76]
*E. coli*	Genome editiong, β-carotene (2.0 g/L) (Central metabolic pathways, MEP pathway)	2015	[77]
*E. coli*	CRISPRi, mevalonate production by *dnaA* (2.1-fold), *oriC* (1.8 fold), *pyrF* (3.5 fold), *thyA* (1.8-fold) deletion	2016	[78]
*E. coli*	CRISPRi, lycopen production in MVA pathway (*mvaK*1, *mvaE*) (9-fold)	2016	[79]
*E. coli*	CRISPRi, Isoprene production in lycopen pathway (*ispA*) (2.6-fold)	2016	[79]
*E. coli*	CRISPRi, 4,4′-dihydroxybiphenyl (4HB) production, P(3HB-*co*-4HB) biosynthesis pathway (*sad1*, *sucD*, *sucC*, *sdhA*, *sdhB*) (13.1-fold)	2015	[80]
*E. coli*	CRISPRi, Pinosylvin, malonyl-CoA pathway (*fabD*) (1.9-fold)	2016	[81]
*E. coli*	CRISPRi, O-methylated anthocyanin, Methionine biosynthetic pathway (*metJ*) (2-fold)	2017	[82]
*E. coli*	CRISPRi, 1,4-Butanediol (1,4-BDO) production, 1,4-BDO biosynthesis pathway (*gabD*, *ybgC*, *tesB*) (1.8 g/L)	2017	[83]
*E. coli*	CRISPRi, Malate production, Glyoxylate pathway (*pyc* from *A. flavus*, *gltA*, *acnB*, *aceA*, *aceB* from *S. coelicolor*) (2.3-fold)	2017	[84]
*E. coli*	CRISPRi, n-butanol production, *pta*, *frdA*, *ldhA*, and *adhE* (3.2 fold)	2017	[85]
*E. coli*	CRISPR, Xylose production, Xylose pathway (*xylA*, *xylB*, *tktA*, *talB*)	2017	[86]
*E. coli*	CRISPRi, Pinosylvin production, Malonyl-CoA pathway (*eno*, *adhE*, *fabB*, *sucC*, *fumC*, *fabF*) (1.7-fold)	2017	[87]
*E. coli*	CRISPRi, Resveratrol production, Malonyl-CoA pathway (*fabD, fabH*, *fabB*, *fabF*, *fabI*) (6-fold)	2017	[88]
*E. coli*	CRISPR, n-butanol production, *gltA*	2017	[89]
